# Design strategy of antidote sequence for bivalent aptamer: Rapid neutralization of high‐anticoagulant thrombin‐binding bivalent DNA aptamer‐linked M08 with HD22

**DOI:** 10.1002/rth2.12503

**Published:** 2021-06-05

**Authors:** Toru Yoshitomi, Koji Wakui, Masato Miyakawa, Keitaro Yoshimoto

**Affiliations:** ^1^ Department of Life Sciences Graduate School of Arts and Sciences The University of Tokyo Tokyo Japan; ^2^ Research Center for Functional Materials National Institute for Materials Science Ibaraki Japan; ^3^ JST PRESTO Tokyo Japan

**Keywords:** anticoagulant, antidote, DNA aptamer, thrombin

## Abstract

**Background:**

Bivalent thrombin‐binding aptamers (TBAs) have great potential for the treatment of thrombosis because they exhibit high anticoagulant activity, and their complementary single‐stranded DNA (ssDNA) sequences work as an antidote. However, a design strategy for antidote sequences against bivalent aptamers has not been established.

**Objectives:**

To develop bivalent TBAs using M08, which exhibits higher anticoagulant activity than the previously reported exosite Ⅰ–binding DNA aptamers, such as HD1, an exosite Ⅱ–binding DNA aptamer (HD22) was linked to M08 with various types of linkers. In addition, short‐length complementary ssDNAs were designed to neutralize the optimized bivalent aptamer effectively and rapidly.

**Results:**

Among the bivalent aptamers of M08 linked to HD22 with various types of linkers, M08‐T15‐HD22 possessed approximately 5‐fold higher anticoagulant activity than previously reported bivalent aptamers. To neutralize the activity of the 87‐meric M08‐T15‐HD22, complementary ssDNA sequences with different lengths and hybridization segments were designed. The complementary sequence against the M08 moiety played a more important role in neutralizing than that against the HD22 moiety. Hybridization of the T15 linker in the M08‐T15‐HD22 with the A15 sequence in the antidote accelerated neutralization due to toehold‐mediated strand displacement. Interestingly, some shorter‐length antidotes showed higher neutralizing activity than the full complementary 87‐meric antidote, and the shortest, 34‐meric antidote, neutralized most effectively.

**Conclusions:**

A pair comprising an 87‐meric bivalent TBA containing M08 and a 34‐meric short‐length antidote with high anticoagulant and rapid neutralizing activities was developed. This design strategy of the DNA sequence can be used for other bivalent DNA aptamers and their antidotes.

**Essentials**

- A new pair comprising an 87‐meric bivalent TBA anticoagulant agent and a 34‐meric ssDNA antidote was developed.
- A bivalent TBA containing M08 with a T15 linker exhibited four times higher activity than M08.
- A short ssDNA antidote with a toehold moiety showed the highest neutralization ability.
- Design strategies for short antidote in the present study can be used for other bivalent aptamers.

## 1 INTRODUCTION

Disseminated intravascular coagulation generated in patients with infectious diseases such as coronavirus disease 2019 is a severe problem. Therefore, the need to develop anticoagulants has been increasing. Simultaneous antidote development is also essential because anticoagulant therapy can be accompanied by side effects such as hemorrhagic complications.

Nucleic acid aptamers, which are single‐stranded DNA (ssDNA) and RNA with high affinity to specific targets such as proteins, could be powerful anticoagulants. Compared with antibodies, DNA aptamers possess many advantages such as relatively small size, greater stability, potential for chemical modification, lower toxicity, and reduced immunogenicity.^1^ Aptamers have been studied as smart biomolecules in numerous investigations for diagnostic^2^ and therapeutic applications.^3^ Recently, we developed the systematic evolution of ligands by exponential enrichment (SELEX) with microbead‐assisted capillary electrophoresis (MACE), referred to as MACE‐SELEX, which features a sophisticated separation step with high sensitivity based on capillary electrophoresis separation using target‐coupled microbeads.^4^ Using the MACE‐SELEX system, we discovered a new thrombin‐binding aptamer (TBA), M08, which exhibits higher anticoagulant activity than a previously reported thrombin exosite I–binding aptamer, HD1.^4^ To improve the activity of DNA/RNA aptamers, an effective strategy using bivalent aptamers has been previously reported.^5^, ^6^, ^7^, ^8^, ^9^ Therefore, in this study, M08 was constructed as a bivalent aptamer by linking to exosite II–binding aptamers such as HD22.

**FIGURE 1:**
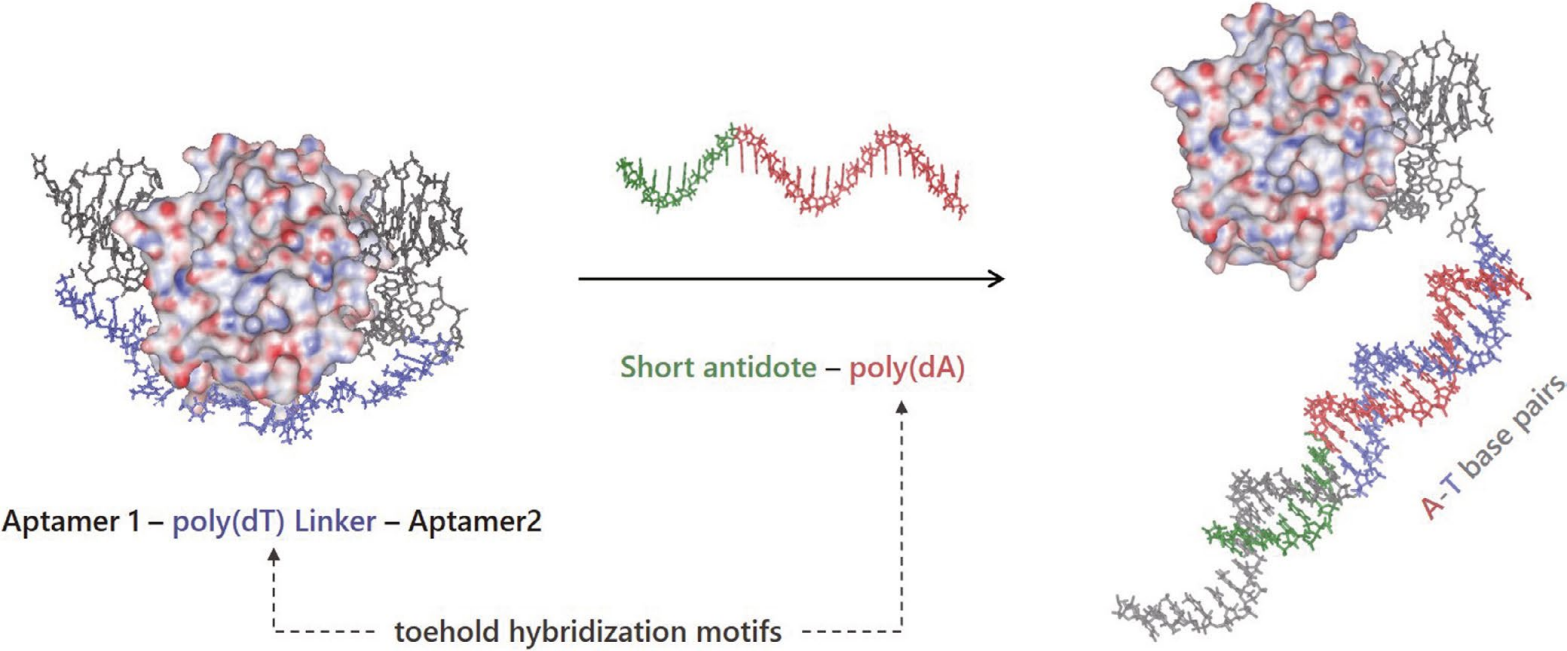
Molecular modeling images of bivalent aptamer dissociation from exosite I on thrombin by short antidote. The radiographic crystallography data of a ternary complex of thrombin with HD1 and HD22 (PDB entry 5EW2) was used as an initial structure. The poly(dT) linker was generated and connected with HD1 and HD22 using HyperChem version 7.5 (Hypercube Inc., USA). Energy minimization was performed with AMBER force field in HyperChem

The activities of aptamers can be reversed by antidotes with reverse complementary sequences.^10^, ^11^ However, a design strategy for antidote sequences against bivalent aptamers has not been established. In the present study, we developed bivalent TBAs using M08, which were linked with thrombin exosite Ⅱ–binding HD22 via poly(dT) or poly(dA) linkers. Remarkably, the anticoagulant activity of M08‐T15‐HD22 was approximately 5‐fold that of the previously reported bivalent TBAs HD1‐A15‐HD22^6^ and HD1‐T16‐HD22.^7^ In addition, to neutralize the activity of the constructed 87‐meric M08‐T15‐HD22, complementary ssDNA sequences with different lengths and hybridization segments were designed, as shown in Figure 1. The most effective and rapid neutralization was accomplished by the shortest, 34‐meric antidote.

## 2 MATERIALS AND METHODS

### 2.1 Chemical reagents

Thrombin from human plasma and all single‐stranded oligonucleotides were purchased from Sigma‐Aldrich (St. Louis, MO, USA). The sequences of oligonucleotides used in this study are listed in Table 1. Fibrinogen and Dulbecco’s phosphate‐buffered saline (PBS) were purchased from FUJIFILM Wako Pure Chemical Corporation (Osaka, Japan). All buffer solutions were prepared using Milli‐Q water (Merck Millipore, Billerica, MA, USA)).

**TABLE 1:** Sequences of oligonucleotides

Name	Sequence (5'→3')
HD1	GGTTGGTGTGGTTGG
HD22	AGTCCGTGGTAGGGCAGGTTGGGGTGACT
M08	AGGTCAGATGATGGGGATGGGGGGTTGGAGGAATGGATGACCT
HD1‐T16‐HD22	GGTTGGTGTGGTTGGTTTTTTTTTTTTTTTTAGTCCGTGGTAGGGCAGGTTGGGGTGACT
HD1‐A15‐HD22	GGTTGGTGTGGTTGGAAAAAAAAAAAAAAAAGTCCGTGGTAGGGCAGGTTGGGGTGACT
M08‐HD22	AGGTCAGATGATGGGGATGGGGGGTTGGAGGAATGGATGACCTAGTCCGTGGTAGGGCAGGTTGGGGTGACT
M08‐A5‐HD22	AGGTCAGATGATGGGGATGGGGGGTTGGAGGAATGGATGACCTAAAAAAGTCCGTGGTAGGGCAGGTTGGGGTGACT
M08‐A10‐HD22	AGGTCAGATGATGGGGATGGGGGGTTGGAGGAATGGATGACCTAAAAAAAAAAAGTCCGTGGTAGGGCAGGTTGGGGTGACT
M08‐A15‐HD22	AGGTCAGATGATGGGGATGGGGGGTTGGAGGAATGGATGACCTAAAAAAAAAAAAAAAAGTCCGTGGTAGGGCAGGTTGGGGTGACT
M08‐A20‐HD22	AGGTCAGATGATGGGGATGGGGGGTTGGAGGAATGGATGACCTAAAAAAAAAAAAAAAAAAAAAGTCCGTGGTAGGGCAGGTTGGGGTGACT
M08‐A25‐HD22	AGGTCAGATGATGGGGATGGGGGGTTGGAGGAATGGATGACCTAAAAAAAAAAAAAAAAAAAAAAAAAAGTCCGTGGTAGGGCAGGTTGGGGTGACT
M08‐T5‐HD22	AGGTCAGATGATGGGGATGGGGGGTTGGAGGAATGGATGACCTTTTTTAGTCCGTGGTAGGGCAGGTTGGGGTGACT
M08‐T10‐HD22	AGGTCAGATGATGGGGATGGGGGGTTGGAGGAATGGATGACCTTTTTTTTTTTAGTCCGTGGTAGGGCAGGTTGGGGTGACT
M08‐T15‐HD22	AGGTCAGATGATGGGGATGGGGGGTTGGAGGAATGGATGACCTTTTTTTTTTTTTTTTAGTCCGTGGTAGGGCAGGTTGGGGTGACT
M08‐T20‐HD22	AGGTCAGATGATGGGGATGGGGGGTTGGAGGAATGGATGACCTTTTTTTTTTTTTTTTTTTTTAGTCCGTGGTAGGGCAGGTTGGGGTGACT
M08‐T25‐HD22	AGGTCAGATGATGGGGATGGGGGGTTGGAGGAATGGATGACCTTTTTTTTTTTTTTTTTTTTTTTTTTAGTCCGTGGTAGGGCAGGTTGGGGTGACT
HD22‐M08	AGTCCGTGGTAGGGCAGGTTGGGGTGACTAGGTCAGATGATGGGGATGGGGGGTTGGAGGAATGGATGACCT
HD22‐A5‐M08	AGTCCGTGGTAGGGCAGGTTGGGGTGACTAAAAAAGGTCAGATGATGGGGATGGGGGGTTGGAGGAATGGATGACCT
HD22‐A10‐M08	AGTCCGTGGTAGGGCAGGTTGGGGTGACTAAAAAAAAAAAGGTCAGATGATGGGGATGGGGGGTTGGAGGAATGGATGACCT
HD22‐A15‐M08	AGTCCGTGGTAGGGCAGGTTGGGGTGACTAAAAAAAAAAAAAAAAGGTCAGATGATGGGGATGGGGGGTTGGAGGAATGGATGACCT
HD22‐A20‐M08	AGTCCGTGGTAGGGCAGGTTGGGGTGACTAAAAAAAAAAAAAAAAAAAAAGGTCAGATGATGGGGATGGGGGGTTGGAGGAATGGATGACCT
HD22‐A25‐M08	AGTCCGTGGTAGGGCAGGTTGGGGTGACTAAAAAAAAAAAAAAAAAAAAAAAAAAGGTCAGATGATGGGGATGGGGGGTTGGAGGAATGGATGACCT
HD22‐T5‐M08	AGTCCGTGGTAGGGCAGGTTGGGGTGACTTTTTTAGGTCAGATGATGGGGATGGGGGGTTGGAGGAATGGATGACCT
HD22‐T10‐M08	AGTCCGTGGTAGGGCAGGTTGGGGTGACTTTTTTTTTTTAGGTCAGATGATGGGGATGGGGGGTTGGAGGAATGGATGACCT
HD22‐T15‐M08	AGTCCGTGGTAGGGCAGGTTGGGGTGACTTTTTTTTTTTTTTTTAGGTCAGATGATGGGGATGGGGGGTTGGAGGAATGGATGACCT
HD22‐T20‐M08	AGTCCGTGGTAGGGCAGGTTGGGGTGACTTTTTTTTTTTTTTTTTTTTTAGGTCAGATGATGGGGATGGGGGGTTGGAGGAATGGATGACCT
HD22‐T25‐M08	AGTCCGTGGTAGGGCAGGTTGGGGTGACTTTTTTTTTTTTTTTTTTTTTTTTTTAGGTCAGATGATGGGGATGGGGGGTTGGAGGAATGGATGACCT
[M08‐T15‐HD22]c	AGTCACCCCAACCTGCCCTACCACGGACTAAAAAAAAAAAAAAAAGGTCATCCATTCCTCCAACCCCCCATCCCCATCATCTGACCT
[M08]c	AGGTCATCCATTCCTCCAACCCCCCATCCCCATCATCTGACCT
[HD22]c	AGTCACCCCAACCTGCCCTACCACGGACT
[M08‐T5]c	AAAAAAGGTCATCCATTCCTCCAACCCCCCATCCCCATCATCTGACCT
[M08‐T10]c	AAAAAAAAAAAGGTCATCCATTCCTCCAACCCCCCATCCCCATCATCTGACCT
[M08‐T15]c	AAAAAAAAAAAAAAAAGGTCATCCATTCCTCCAACCCCCCATCCCCATCATCTGACCT
[M08s‐T15]c	AAAAAAAAAAAAAAAAGGTCATCCATTCCTCCAA

### 2.2 Evaluation of the anticoagulant activities of TBAs

To evaluate anticoagulant activity, clotting time was measured using a microplate reader (Viento Nano; BioTek Japan, Tokyo, Japan). The clotting curve was measured using the absorbance at 350 nm associated with fibrin gel formation. After annealing at 95°C for 2 minutes, the samples were slowly cooled to 25°C at a rate of 0.1°C/s. Twenty microliters of each 10 µM TBA and 20 μL of 1 µM thrombin were added to 280 μL of PBS (pH = 7.4) and incubated for 15 minutes at 25°C. Then, 20 μL of 2 mg/mL fibrinogen was added to 80 μL of the reaction mixture. Absorbance measurements were initiated after the sample was mixed. The final concentrations of thrombin, TBA, and fibrinogen were 2.5 nM, 2.5 nM, and 0.4 mg/mL, respectively. The reaction of thrombin and fibrinogen was always conducted in parallel with other samples as internal standards; all clotting times were normalized based on the internal standard. The clotting time was calculated according to a previously described procedure.^12^

### 2.3 Evaluation of the neutralizing activities of antidotes

To evaluate the neutralizing activity of antidotes, a clotting time assay was used. In summary, 10 μL of each 200‐nM TBA and 10 μL of 100‐nM thrombin were added to 180 μL of PBS (pH = 7.4) and incubated for 15 minutes at 25°C. Then, 50 μL of the mixture with 0.8 mg/mL of fibrinogen and 10 nM of antidote was added to 50 μL of the reaction mixture. Absorbance measurements were initiated after mixing the sample. The final concentrations of thrombin, TBA, antidote, and fibrinogen were 2.5 nM, 5 nM, 5 nM, and 0.4 mg/mL, respectively. The reaction of thrombin, M08‐T15‐HD22, and fibrinogen was always conducted in parallel with other samples as internal standards; all clotting times were normalized based on the internal standard.

## 3 RESULTS AND DISCUSSION

**FIGURE 2:**
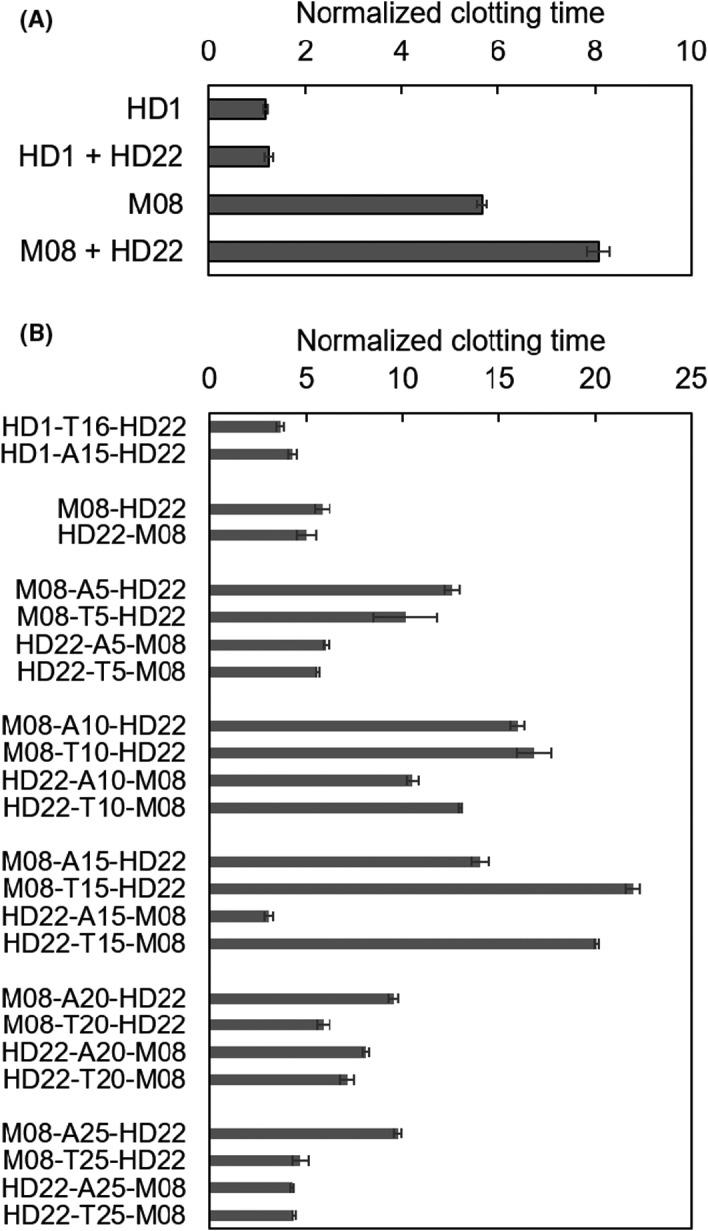
Anticoagulant activities of monovalent aptamers HD1 and M08, their mixture with HD22, and their bivalent aptamers with different length of linker. A, The normalized clotting time of fibrinogen after mixing with thrombin in the presence of HD1, M08, or its mixture with HD22. B, The normalized clotting time of fibrinogen after mixing with thrombin in the presence of bivalent aptamers comprising linked M08 and HD22 with different‐length linkers. A reaction of thrombin and fibrinogen was used as an internal standard and its clotting time was defined as 1. Final concentrations: aptamer = 2.5 nM, thrombin = 2.5 nM, and fibrinogen = 0.4 mg/mL

In a previous study using MACE‐SELEX, we discovered an M08 aptamer that formed an antiparallel or hybrid quadruplex structure.^4^ M08 probably interacts with exosite I of thrombin, similar to previously reported exosite I–binding HD1,^4^ because M08 had approximately 5‐fold higher anticoagulant activity than the previously reported HD1 under the present conditions (Figure 2A). First, we investigated the synergistic effect of two aptamers that bind to exosite I or II^13^ using HD1, M08, and HD22. Under the experimental conditions, the clotting time of HD1 mixed with HD22 (HD1 + HD22) was slightly prolonged, approximately 1.05 times compared to HD1 alone (Figure 2A). In contrast, the mixture of M08 and HD22 (M08 + HD22) prolonged the clotting time by approximately 1.3 times compared to M08 alone (Figure 2A). It should be noted that the mixture of M08 and HD22 prolonged the clotting time by approximately 6.5 times compared to the mixture of HD1 and HD22 (Figure 2A). These increases in affinity and anticoagulant activity of M08 resulted from synergistic effects with HD22.

To improve the anticoagulant activity of M08 further, we constructed bivalent aptamers of M08 linked to HD22 via various types of linkers. In a previous report, poly(dA) and poly(dT) linkers were selected to construct the bivalent aptamers HD1‐A15‐HD22,^6^ and HD1‐T16‐HD22 ^7^ due to their weak interactions with the G‐quadruplex moieties of the individual aptamers. A previous study reported that the anticoagulant activities of HD1‐22 are nearly identical to those of bivalirudin and superior to those of argatroban.^11^ We investigated the effect of both poly(dA) and poly(dT) linkers on the anticoagulant activities of M08‐containing bivalent aptamers. Figure 2B shows the activities of bivalent aptamers containing HD1, M08, and HD22 with various types of linkers. As reported previously, the anticoagulant activities of HD1‐A15‐HD22^6^ and HD1‐T16‐HD22^7^ (Figure 2B) were higher than those of HD1 mixed with HD22 (Figure 2A), where the A15 linker was more effective than the T16 linker. M08‐containing bivalent aptamers linked to HD22 were constructed using A5, A10, A15, A20, A25, T5, T10, T15, T20, and T25 linkers. M08‐containing bivalent aptamers without a linker (M08‐HD22 and HD22‐M08) exhibited lower anticoagulant activity than M08 mixed with HD22, while more than half of the bivalent aptamers having linkers exhibited higher anticoagulant activity than M08 mixed with HD22. Among the constructed bivalent aptamers, M08‐T15‐HD22 showed the highest anticoagulant activity, approximately four times that of monomeric M08. It should be noted that the T15 linker in M08‐T15‐HD22 extended the clotting time by approximately 1.4 times that of the A15 linker in M08‐A15‐HD22. These results suggested that poly(dT) linkers are more suitable than poly(dA) linkers for constructing bivalent TBA using M08 and HD22. Intriguingly, the optimal length of poly(dT) linkers was different from that of poly(dA); 10‐mers and 15‐mers were optimal for poly(dA) and poly(dT) linkers, respectively. Compared to poly(dT), poly(dA) has a less flexible polymer chain owing to the strong stacking interactions between purine bases.^14^ This may be the reason for the difference between poly(dA) and poly(dT) linkers. In addition, the configuration of the two aptamers with a linker in a bivalent aptamer also affected the anticoagulant activities. Their activities tended to be higher when M08 was located at the 5′ end and HD22 was located at the 3′ end (Figure 2B). A previous study also reported that the activity was higher when HD1 was located at the 5′ end and HD22 was located at the 3′ end due to steric constraints.^11^ M08‐T15‐HD22 has a 4 to 5 times longer clotting time than the previously reported bivalent TBAs HD1‐A15‐HD22^6^ and HD1‐T16‐HD22,^7^ indicating that M08‐T15‐HD22 is the most potent bivalent TBA anticoagulant reagent at present.

**FIGURE 3:**
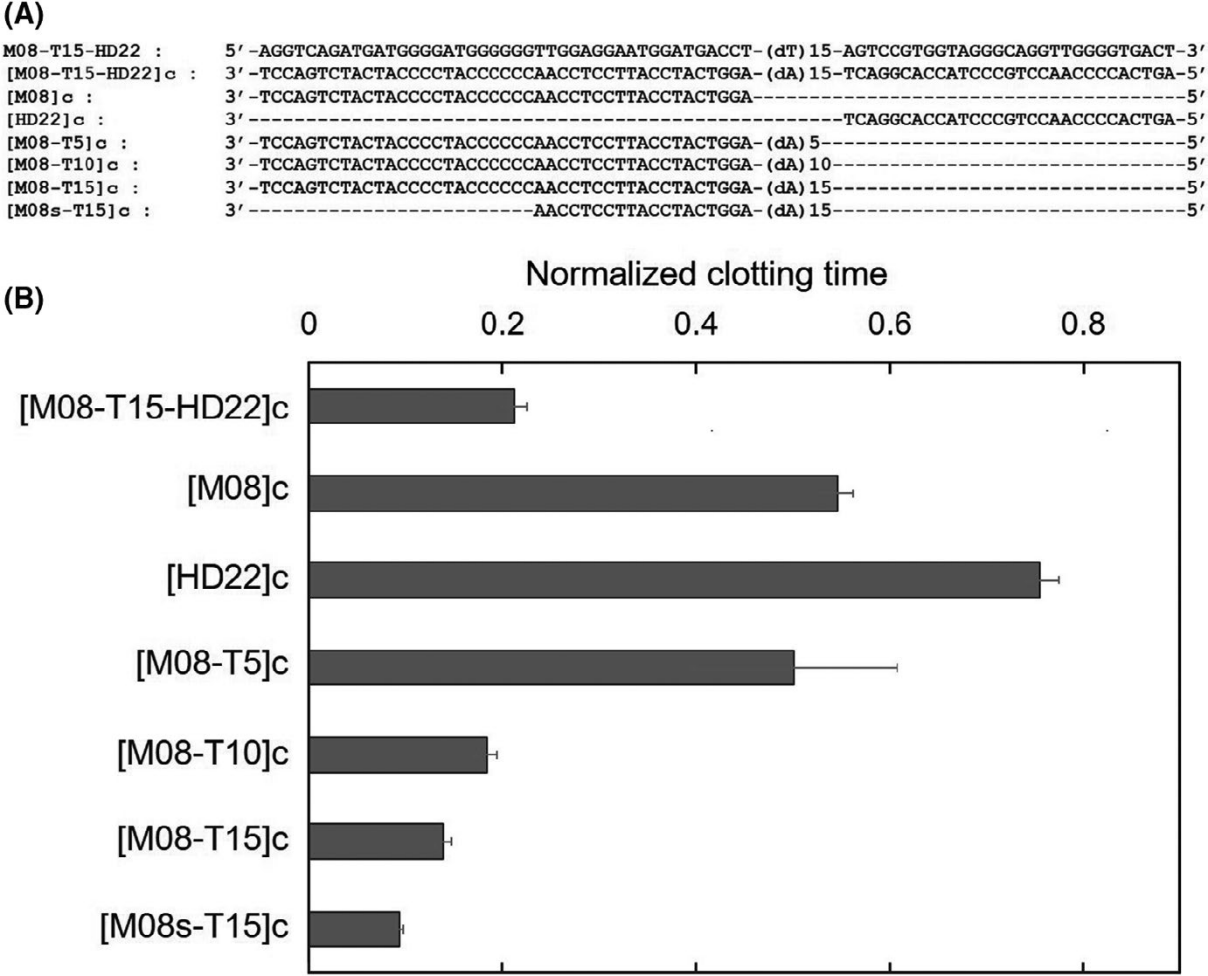
Neutralizing ability of antidotes against M08‐T15‐HD22. A, Sequences of M08‐T15‐HD22 and all tested antidotes. B, The normalized clotting time of fibrinogen after mixing thrombin and M08‐T15‐HD22 in the presence of complementary single‐stranded DNA as the antidote. A reaction of thrombin, M08‐T15‐HD22, and fibrinogen was used as an internal standard and its clotting time was defined as 1. Final concentrations: aptamer = 5 nM, thrombin = 2.5 nM, fibrinogen = 0.4 mg/mL, and antidote = 5 nM

Anticoagulant therapy can be accompanied by hemorrhagic complications.^15^ The greatest advantage of anticoagulant aptamers is the ability to neutralize their pharmacological activity using complementary ssDNAs.^10^ However, a design strategy for antidote sequences against bivalent aptamers has not been reported. To reveal the important moiety in antidote ssDNA, the changes in clotting times by the addition of several complementary ssDNAs were measured. Seven types of antidote ssDNAs, shown in Figure 3A, were designed and added to a mixture consisting of thrombin, M08‐T15‐HD22, and fibrinogen to evaluate their antidote effects. In Figure 3B, the normalized clotting times are expressed as the value on the *y* axis relative to that of a mixture consisting of thrombin with M08‐T15‐HD22 and fibrinogen. [M08‐T15‐HD22]c, which is the complementary ssDNA of full‐length M08‐T15‐HD22, shortened the relative clotting time to 0.21. In contrast, the short complementary ssDNA of each exosite I– and II–binding aptamers, [M08]c and [HD22]c, shortened the clotting time to 0.54 and 0.75, respectively. The neutralization efficacy of [M08]c was higher than that of [HD22]c. This indicates that [M08]c, the complementary ssDNA moiety against the exosite I–binding aptamer, is needed for the rapid neutralization of anticoagulant bivalent TBAs. This is probably due to the fact that fibrinogen binds to exosite I in thrombin.^16^

The additional poly(dA) moiety in antidote ssDNA plays an important role in effective neutralization. [M08]c with different lengths of poly(dA), namely, [M08‐T5]c, [M08‐T10]c, and [M08‐T15]c, showed shorter clotting times than [M08]c, and their clotting times depended on the length of poly(dA). These results indicate that poly(dA) functioned as a toehold moiety for effective DNA hybridization.^17^ There is a high energy barrier in the hybridization of [M08]c with the M08 moiety in the bivalent aptamer because of the large conformational change of the aptamer from a highly ordered structure to double‐stranded DNA. The additional poly(dA) moiety in the antidotes easily accesses and hybridizes with a single‐stranded poly(dT) linker in a bivalent aptamer without any conformational change. Interestingly, the clotting times of 58‐meric [M08‐T15]c and 34‐meric [M08s‐T15]c were 0.13 and 0.094, respectively, which were shorter than that of 87‐meric [M08‐T15‐HD22]c. These results indicate that antidote ssDNA for bivalent aptamers should include complementary ssDNA against the linker moiety. In addition, the complementary ssDNA moiety against the exosite I–binding aptamer can be shortened to at least the length that releases the exosite I–binding aptamer from thrombin. The molecular modeling image for the neutralization of bivalent TBA by short antidote ssDNA is shown in Figure 1. Short antidote ssDNA hybridized effectively with the poly(dT) linker in the bivalent aptamer and released the exosite I–binding aptamer from thrombin. Release of the exosite II–binding aptamer from thrombin is less important to increase the neutralizing ability of the antidote ssDNA.

## 4 CONCLUSION

In summary, we succeeded in developing an effective anticoagulant 87‐meric bivalent DNA aptamer, M08‐T15‐HD22, and its 34‐meric antidote ssDNA, [M08s‐T15]c. This bivalent approach exhibited approximately four times higher anticoagulant activity than monomeric M08. Furthermore, M08‐T15‐HD22 displayed the highest anticoagulant activity among the bivalent TBAs currently reported. Interestingly, the shortest antidote [M08s‐T15]c showed a higher neutralizing ability than the long‐antidote [M08‐T15‐HD22]c, which is the fully complementary ssDNA of M08‐T15‐HD22. Since shortening of the antidote decreases dosage mass, the development of a low‐molecular‐weight antidote ssDNA is an important factor in successful clinical trials. In the present study, we found important guidelines for the design of short‐antidote ssDNAs against anticoagulant bivalent TBAs. For the rapid and effective neutralization of anticoagulant bivalent TBAs, a complementary sequence against the exosite I–binding aptamer in the bivalent aptamer is needed in the antidote ssDNA, which can be shortened to at least the length that releases the exosite I–binding aptamer from thrombin. In addition, complementary sequences against the linker moiety in bivalent aptamers play an important role in increasing the neutralizing ability of antidote ssDNA, where antidote ssDNAs with longer complementary sequences showed higher neutralizing ability than those with shorter sequences. Because the complementary ssDNA moiety against exosite II–binding aptamer is less important for the neutralization of a bivalent aptamer, the antidote does not need to include a complementary ssDNA moiety against the exosite II–binding aptamer. Thus, short‐antidote ssDNAs can be constructed using the sequence information of the exosite I–binding aptamer and the linker in bivalent aptamers.

In recent years, various bivalent aptamers with longer chains have been reported to show higher bioactivity and mimic the biological activity of natural proteins,^18^, ^19^ and they have great potential as new‐generation drugs. Since rapid neutralization by antidotes with low dosage mass is crucial for the use of bivalent DNA aptamers in clinical treatment, the experimental results and conclusions contained in this study could support development of ssDNA sequences for neutralizing bivalent aptamers.

## RELATIONSHIP DISCLOSURE

This study was partially funded by Nissan Chemical Cooperation, who had no control over the interpretation, writing, or publication of this work.

## AUTHOR CONTRIBUTIONS

TY analyzed data and wrote the manuscript. KW and MM performed research and analyzed data. KY designed and coordinated research, analyzed data, and wrote the manuscript.
